# The *Latest Time Point of Retreatment* (LTPR) as a Novel Method to Determine Antibacterial Effects for Binary Use of Cold Atmospheric Plasma and Conventional Agents

**DOI:** 10.3389/fmicb.2020.576500

**Published:** 2020-10-29

**Authors:** Sandra Schramm, Karl-Anton Hiller, Sylvia Cantzler, Hannes Weilemann, Maximilian Cantzler, Julia L. Zimmermann, Fabian Cieplik, Tim Maisch

**Affiliations:** ^1^Department of Dermatology, University Hospital Regensburg, Regensburg, Germany; ^2^Department of Conservative Dentistry and Periodontology, University Hospital Regensburg, Regensburg, Germany; ^3^Terraplasma GmbH, Garching, Germany

**Keywords:** LTPR, cold atmospheric plasma, biofilm, binary combination, antibacterial effects

## Abstract

Multi-resistant microorganisms are a long-standing problem for public healthcare, as inactivating those resistant pathogens with conventional antibiotics or antiseptics often no longer achieves the expected clinical success. The aim of this *in vitro* study was to investigate the antibacterial efficacy of binary combinations of conventional antibacterial agents with cold atmospheric plasma (CAP), when both are applied in non-lethal concentrations. In this study, *Enterococcus faecalis* biofilms were treated with CAP in binary combinations with benzalkonium chloride (BAC), chlorhexidine (CHX), or ciprofloxacin (CIP), respectively, which were applied in different sequences. In order to evaluate effects of binary use of two different antibacterial approaches, the so-called *latest time point of retreatment* (LTPR) was defined. For this purpose, regrowth curves of the bacteria were measured following the respective treatment combinations. LTPR is defined as the time component of the inflection point of a normalized regrowth curve and allows the rating and interpretation of single or binary treatments with different agents or approaches. Furthermore, LTPR designates the latest time point where a retreatment appears to be appropriate for preventing regrowth of the bacteria in case the first treatment was not lethal. Here in our study, the binary combination of 10 min CAP with BAC, CHX, or CIP leads to higher LTPRs as compared to single treatments for both sequences of application. Overall, the combination of two antimicrobial approaches is an effective alternative for inactivating bacteria in biofilms instead of a single treatment. Thus, LTPR provides a novel promising way to determine antibacterial effects for single or binary use of given antimicrobial approaches.

## Introduction

The emergence of multi-resistant microorganisms is a growing problem in medicine, as there are hardly any successful antimicrobial techniques for inactivating those resistant pathogens. The development of new antimicrobial substances is stagnating and conventional methods do not show sufficient antibacterial effects in these cases (Rossolini and Mantengoli, 2008). Antibiotic resistance and the resulting threats of nosocomial infections are often associated with the presence of Gram-positive enterococci (Portenier et al., 2003).

As early as in 1990, Murray recognized that due to the increasing development of bacterial resistance, the treatment of enterococcal infections was becoming more and more of a challenge (Murray, 1990). Especially *Enterococcus faecalis* frequently causes infections such as urinary tract infections, wound infections, bacteremia, intra-abdominal infections and endocarditis (Moellering, 1992; Portenier et al., 2003). *E. faecalis* is also present in the resident oral microbiota of healthy people, but its relative numbers are quite small. However, as *E. faecalis* is the main taxa found in secondary endodontic infections, it could be assumed that its presence in the oral cavity may be at a larger number than assumed in previous studies (Portenier et al., 2003).

One of the reasons why the elimination of pathogens such as *E. faecalis* is very difficult is because they are able to produce biofilms attached to surfaces that are much more tolerant toward antimicrobial approaches than their planktonic counterparts (Joyanes et al., 2000; Römling and Balsalobre, 2012; Theinkom et al., 2019). Biofilm formation is found in up to 80% of bacterial infections in humans (Römling and Balsalobre, 2012). The complex structure of biofilms protects the bacteria they contain from external influences (Costerton et al., 1999; Donlan and Costerton, 2002). Many reasons are discussed why bacteria in biofilms are more tolerant than planktonic bacteria. The biofilm matrix (extracellular polymeric substances; EPS) serves as a physical barrier and delays or prevents the penetration of antimicrobial substances (Mah and O’Toole, 2001; Stewart and William Costerton, 2001). In addition, the heterogeneity of the cells in the biofilm can lead to certain parts being more and others less protected (Mah and O’Toole, 2001). Finally, external environmental influences can lead to the development of specific temporary resistant phenotypes, so called persister cells (Mah and O’Toole, 2001). Therefore, new antimicrobial treatment approaches are needed that (1) are capable of inactivating resistant pathogens not only in planktonic cultures, but also in biofilms and (2) do not themselves pose the risk of inducing new resistances.

In this respect, the use of cold atmospheric plasma (CAP) may open up new options. CAP is referred to as the fourth state of matter in addition to solid, liquid and gaseous. If energy is supplied to a gas, ions and free electrons are created. The so-called ionized gas (Niemira, 2012) contains a mixture of electrons, protons, ions, excited atoms and molecules, reactive oxygen species (such as O_3_, O^2–⁣⋅^, H_2_O_2_, HO^⋅^), reactive nitrogen species (such as ^⋅^NO, NO_2_, ONOO^–^) and photons (from the UV and visible wavelength range) (Stoffels et al., 2008; Maisch et al., 2012c). There are several ways in which CAP can be produced. Using the dielectric barrier discharge (DBD) technology, the contaminated surface itself serves as a counter-electrode for the generation of CAP, whereas CAP is generated indirectly and in ambient air and diffuses to the contaminated surface when employing a surface micro-discharge (SMD) plasma technology. The latter therefore is a contact-free process facilitating the use of CAP *in vivo* (Maisch et al., 2012a). It has been shown that CAP treatment can reduce the bacterial load in chronic wounds and also has beneficial effects on wound healing (Isbary et al., 2010; Eggers et al., 2020). Furthermore, treatment of chronic wounds by CAP is painless and has no side effects on the surrounding tissue (Isbary et al., 2010; Maisch et al., 2012b), because even the formation of UV radiation at atmospheric pressure is of such low extent that neither eukaryotic nor bacterial cells are damaged (Klämpfl et al., 2012; Maisch et al., 2012c). Therefore, the antimicrobial effects of CAP are not due to UV radiation but rather due to the emergence of reactive oxygen and nitrogen species (Maisch et al., 2012a,b).

Cold atmospheric plasma is known to be effective against bacteria, fungi, and spores (Klämpfl et al., 2012) and it has also been shown that it can be successfully used to inactivate *E. faecalis* in biofilms (Li et al., 2015). In addition, according to the current state of research, bacteria do not develop resistance to CAP treatment, even after repeated application (Brun et al., 2018).

Benzalkonium chloride (BAC), chlorhexidine (CHX) and ciprofloxacin (CIP) are often used as antimicrobial agents in clinical practice. BAC and CHX are antiseptics, frequently used for disinfection of skin or in the oral cavity, with both having a similar membrane-disruptive mechanism of action by binding to negatively charged bacterial cell surfaces due to their positive charge (Botelho, 2000; Gilbert and Moore, 2005; Nomura et al., 2010; Muehler et al., 2017; Cieplik et al., 2019). It has already been shown that both, BAC and CHX, exert antibacterial effects against *E. faecalis* (Zhang et al., 2015; Baron et al., 2016). CIP is an antibiotic from the fluoroquinolone group, whose antibacterial effect is based on the inhibition of the enzyme DNA gyrase (Ball, 1986; Brun et al., 2018).

Although these results are promising, it is not always possible to completely eliminate all bacteria, including resistant bacteria or those in persistent infections with just one antimicrobial approach. Therefore, the combination of CAP with conventional antimicrobial agents could be an interesting option. For instance, Herbst et al. (2015) showed that CHX showed significantly stronger antibacterial effects in infected root canals when combined with CAP than CAP or CHX alone.

Other studies have already investigated combinations of different antimicrobial agents. In their 2018 review, Wozniak and Grinholc summarized the combination of antimicrobial photodynamic therapy (aPDT) with other antimicrobial agents. It was concluded that a combination of two therapies generally leads to a greater antimicrobial effect than monotherapies. In addition, the duration of the respective treatment can be shortened and the risk of inducing resistances may be reduced, since smaller amounts and lower concentrations, e.g., of antibiotics are used (Wozniak and Grinholc, 2018; Nakonieczna et al., 2019). Ronqui et al. (2016) investigated in 2016 whether the combination of CIP with aPDT led to synergistic effects compared to the effect with aPDT only. For this purpose, experiments with planktonic *Staphylococcus aureus* and *Escherichia coli* were performed, including different treatment sequences of aPDT and CIP. They combined sub-inhibitory doses of both, aPDT and CIP, in order to detect synergistic effects. They found an increased bacterial reduction, especially when aPDT was applied first and followed by CIP (Ronqui et al., 2016). In a further study synergistic effects were shown when combining aPDT and oxacillin on both, planktonic bacteria and biofilms (Iluz et al., 2018).

As those studies investigated the effect of the combination of conventional antimicrobials with aPDT, similar effects might also be achieved when combining CAP with conventional antimicrobials. Therefore, the aim of this study was to investigate the effect on antibacterial efficacy when binary combinations of non-lethal doses of CAP are applied with non-lethal doses of BAC, CHX or CIP. In order to evaluate those effects of binary use of two different antibacterial approaches, the so-called *latest time point of retreatment* (LTPR) was defined and used for rating and interpretation of antibacterial effects.

## Materials and Methods

### Thin-Film Surface Micro-Discharge Plasma Source

**FIGURE 1:**
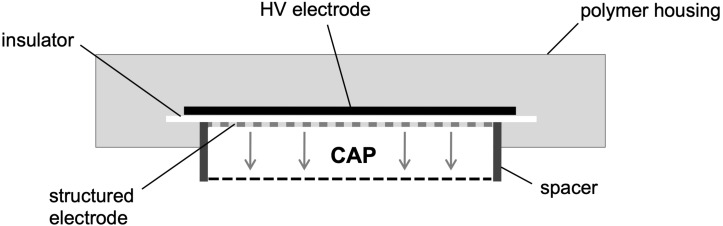
Scheme is depicting the prototype of the surface micro-discharge (SMD) plasma source. By applying a high sinusoidal AC voltage of 3.5 kV_PP_ and a frequency of 4.0 kHz (power consumption of 0.5 to 1 W) under ambient conditions, CAP is produced at the structured electrode and then diffuses to the sample. This scheme was adopted and modified from Theinkom et al. (2019).

In this study, a prototype of a CAP source was used as it has been described earlier in detail (Theinkom et al., 2019). In short, the integrated surface micro-discharge plasma source consists of a high voltage electrode, an insulator plate and a structured electrode and is surrounded by a plastic case (Figure 1). For the treatment under ambient conditions, the plasma source is placed over the sample. By applying a high sinusoidal AC voltage of 3.5 kV_PP_ and a frequency of 4.0 kHz (power consumption of 0.5 to 1 W), CAP is produced at the structured electrode and then diffuses to the sample. The circular, structured electrode is surrounded by a spacer that keeps the plasma inside.

### Compounds

Benzalkonium chloride, chlorhexidine, and ciprofloxacin were purchased from Sigma-Aldrich (St. Louis, MO, United States) and serial dilutions were prepared yielding the following concentrations. For BAC 0.36, 0.18, 0.09, 0.05, and 0.02 mg/mL were used, for CHX 2.12, 1.06, 0.53, 0.27, 0.13, and 0.07 mg/mL and for CIP 1, 0.5, 0.25, and 0.13 mg/mL. BAC was diluted in phosphate-buffered saline (PBS; Dulbecco’s Phosphate Buffered Saline; Sigma-Aldrich, St. Louis, MO, United States), CHX and CIP in *aqua dest*.

### Biofilm Culture

*Enterococcus faecalis* (ATCC 4083) was obtained from the ATCC (American Type Culture Collection, Manassas, VA, United States). Bacteria were grown and maintained on Brain Heart Infusion (BHI) agar plates (provided by the Institute of Clinical Microbiology and Hygiene, University Hospital Regensburg, Germany). For preparation of planktonic cultures, colonies were picked, suspended in 5 mL BHI broth (Sigma-Aldrich) and incubated aerobically over-night to obtain bacteria in the stationary growth phase. Afterward, cultures were harvested by centrifugation and the optical density (OD) was adjusted to 0.6 in BHI broth, as measured at 600 nm by a photometer (SPECORD 50 Plus, Analytik Jena, Jena, Germany). Biofilms were formed in cell culture dishes (Primaria Easy Grip Cell Culture Dish, Corning Inc., Corning, NY, United States) by adding 1 mL fetal calf serum (FCS; Pan-Biotech GmbH, Aidenbach, Germany) and 1 mL of the bacterial suspension (OD = 0.6) for each dish, under spinning conditions to ensure that the bottom of the dishes was evenly covered. Then, the dishes were incubated aerobically at 37°C for 24 h.

### Biofilm Experiments With CAP, BAC, CHX, and CIP Against *E. faecalis*

After the biofilm culture period of 24 h, the supernatants were carefully discarded before the respective treatment. Biofilms were treated for 5 (BAC), 1 (CHX) or 10 min (CIP) with 1 mL each. Biofilms prepared for CAP treatment were first dried for 30 min in a lamina flow before treatment for 0, 1, 3, 5, or 10 min, whereby the CAP device was placed over the sample.

### Range Finding Experiments

For determining non-lethal concentrations of CAP, BAC, CHX, and CIP alone, biofilms were treated with CAP for the several CAP treatment periods and with BAC, CHX, and CIP in their different concentrations.

After the respective treatments, BAC, CHX or CIP were carefully discarded and 1 mL of PBS was added to each biofilm before the biofilm was removed from the bottom of the dish by using a cell scraper (Cell scraper 25 cm, Sarstedt, Newton, NC, United States) and resuspended. 50 μL of each biofilm dissolved in PBS and 450 μL BHI broth were added to two wells of a 48-well plate and mixed thoroughly. After that, the OD of the two separate wells was measured at 600 nm every 30 min from 0 to 180 min using the VarioSkan Flash (SkanIt v. 2.4.5, Thermo Fisher Scientific, Vantaa, Finland). In between, the plates were incubated at 37°C under aerobic conditions without shaking.

The resulting regrowth curves of these range finding experiments are depicted in Supplementary Figure S1. BAC 0.36 mg/mL as well as CHX 1.06 and 2.12 mg/mL led to no detectable regrowth of the bacteria, all other concentrations or treatment periods led to delayed bacterial regrowth. Therefore, 0.18 mg/mL, 0.27 mg/mL, or 1 mg/mL were determined as non-lethal concentrations for BAC, CHX or CIP, respectively, and used for further combination experiments.

### Combination Experiments

In the combination experiments, binary combinations of each CAP treatment period with the selected non-lethal concentration of all compounds were evaluated. The experiments were carried out as combined binary applications of either BAC, CHX or CIP treatment first followed by CAP treatment (sequence 1) or vice versa (sequence 2). In addition, as internal controls, single applications of CAP treatment periods (0 mg/mL BAC, CHX, or CIP) and of BAC, CHX or CIP treatment only (0 min CAP) were performed. After the respective treatments, biofilms were resuspended and regrowth curves were measured as described above every 30 min for 3 h.

### Data Analysis

In all cases BHI broth with PBS alone (blank values) was used as internal control in duplicate. Original results of the antibacterial assays were derived as OD values, corrected with corresponding blank values. Medians and neighboring quartiles were calculated from six independent experiments, each performed in duplicate.

For the range finding experiments, these values were depicted as regrowth curves (OD by measurement time; exemplarily shown in Supplementary Figure S1). To determine non-lethal concentrations of BAC, CHX, and CIP for the combination experiments, these regrowth curves were used. Here in our study, a non-lethal concentration is defined as a concentration that showed delayed regrowth compared to the untreated control but did not show a lethal effect during a 3 h period after application. A concentration is defined as lethal if no regrowth is detectable during the measurement period.

**FIGURE 2:**
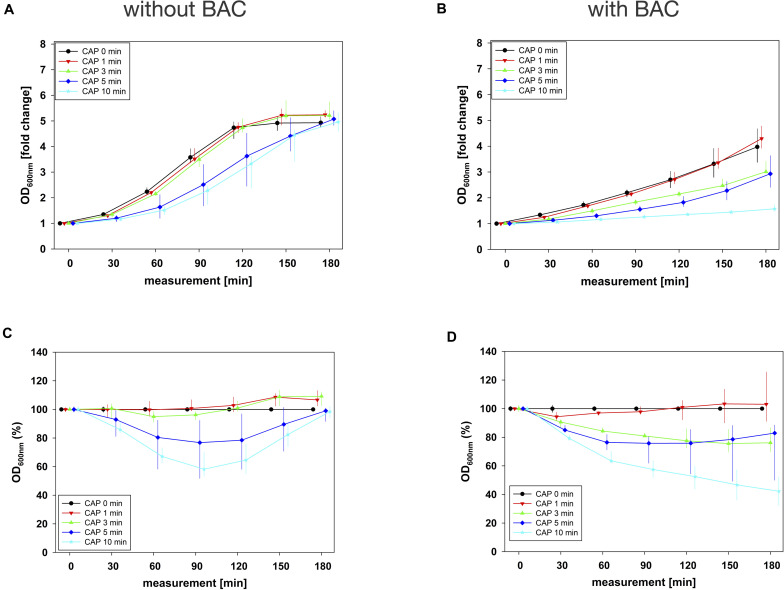
Antibacterial assay against *E. faecalis* biofilms with CAP alone and in binary combination with BAC first (sequence 1). Several CAP treatment periods were applied to *E. faecalis* biofilms and OD was measured at 600 nm every 30 min from 0 to 180 min after treatment. For each CAP treatment period, the values of measurement time = 0 were set to 1, corresponding following values were related to this and depicted as fold change. The results of the OD as a function of the measurement time were displayed as normalized regrowth curves **(A,B)**, those depicted in A were treated with CAP only, those in B were additionally treated with BAC (0.18 mg/mL, 5 min) first (sequence 1). These OD values were related to matching CAP-untreated samples for each measurement time (0 min CAP, black circles), expressed as percentages and depicted without BAC **(C)** and with BAC **(D)** as CAP-normalized regrowth curves. All results were depicted as medians with quartiles of six independent experiments in duplicate.

For the combination experiments, several CAP treatment periods were applied to *E. faecalis* biofilms in binary combination with one non-lethal concentration of BAC, CHX, or CIP. OD was measured at 600 nm every 30 min from 0 to 180 min after the treatment. For each CAP treatment period, the values of measurement time 0 min were set to 1 and the values of the following measurement times were related to this and depicted as fold change. The results of the OD as a function of the measurement time were displayed as normalized regrowth curves (exemplarily shown in Figures 2A,B).

These normalized regrowth curves, expressed as fold changes, were related to matching CAP-untreated samples for each measurement time (0 min CAP), expressed as percentages and depicted as CAP-normalized regrowth curves (exemplarily shown in Figures 2C,D).

Values of the CAP-normalized regrowth curves were put into a general 2-dimensional fit using the correlation coefficient r^2^ as a measure for the goodness of fit, resulting in typical dose-response curves (exemplarily shown in Supplementary Figure S5). The dose-response curves were generated using standard procedures including the sum of the least squares method implemented in the generally available software package TableCurve (TableCurve 2D v. 5.01; Systat Software Inc., San Jose, CA, United States) during curve fitting. The time components of the first inflection points including 95% confidence intervals were derived, statistically evaluated applying the method of Tukey’s intervals and defined as *latest time point of retreatment* (LTPR; shown in Table 1 and Figure 5).

**TABLE 1:** Latest time point of retreatment (LTPR) derived from CAP-normalized regrowth curves.

			CAP [min]			
Sequence	Agent		1	3	5	10
1	BAC	No	−5^§^	−5^§^	42 (30–58)	42 (29–59)
		Yes	−5^§^	181^#^	49 (nd-85)	181^#^
	CHX	No	29 (18–43)	32 (nd)	34 (nd-62)	36 (32–41)
		Yes	−5^§^	−5^§^	69 (56–82)	181^#^
	CIP	No	−5^§^	61 (57–65)	−5^§^	45 (37–53)
		Yes	29 (19–44)	39 (35–42)	180 (180–180)	180 (180–180)
2	BAC	No	−5^§^	−5^§^	48 (32–67)	42 (33–53)
		Yes	180 (180–180)	181^#^	5^§^ (5–5)	181^#^
	CHX	No	−5^§^	5^§^ (5–5)	5^§^ (5–5)	51 (39–65)
		Yes	67 (58–76)	180 (180–180)	180 (180–180)	181^#^
	CIP	No	−5^§^	−5^§^	53 (36-nd)	53 (43–63)
		Yes	32 (nd)	42 (36–50)	33 (23–43)	180 (180–180)

*nd, not determinable.^§^ no LTPR determinable.^#^ no regrowth detectable. Values of the CAP-normalized regrowth curves were fitted (resulting typically in dose response curves, Supplementary Figure S5), the first inflection points including 95% confidence intervals were derived and defined as LTPRs (min). Negative values mean that the graph runs like the reference line (0 min CAP), small positive values (5 min) mean that the corresponding fit was waveform shaped and therefore, in both cases no LTPRs were determinable. The LTPR at 180 min means that the graph remains at its minimum level. If the LTPR is depicted later than 180 min, the graph decreases even further and no regrowth is detectable. Values of this table were repeatedly depicted as Figure 5, including results of statistical analyses.*

All analyses were performed using SPSS for Windows, v. 25 (SPSS Inc., Chicago, IL, United States), except curve fitting, which was done by using TableCurve.

## Results

### Combination Experiments

**FIGURE 3:**
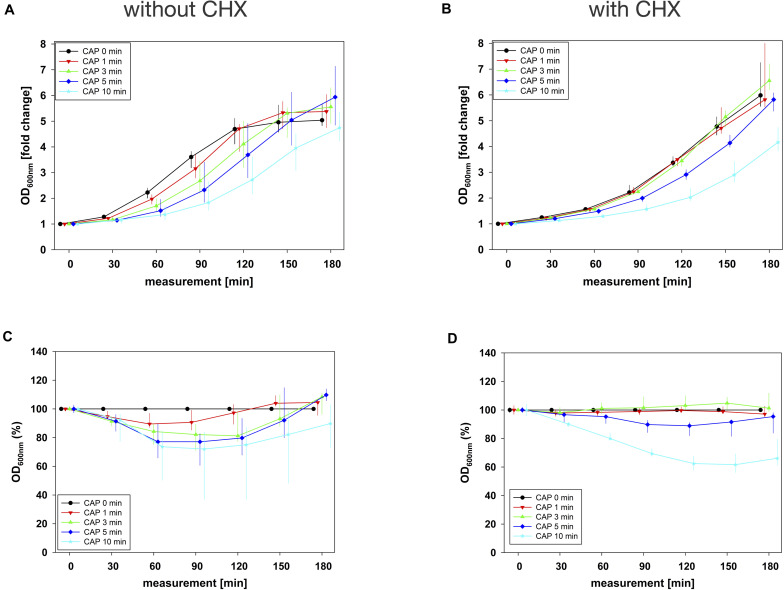
Antibacterial assay against *E. faecalis* biofilms with CAP alone and in binary combination with CHX first (sequence 1). Several CAP treatment periods were applied to *E. faecalis* biofilms and OD was measured at 600 nm every 30 min from 0 to 180 min after treatment. For each CAP treatment period, the values of measurement time = 0 were set to 1, corresponding following values were related to this and depicted as fold change. The results of the OD as a function of the measurement time were displayed as normalized regrowth curves **(A,B)**, those depicted in A were treated with CAP only, those in B were additionally treated with CHX (0.27 mg/mL, 1 min) first (sequence 1). These OD values were related to matching CAP-untreated samples for each measurement time (0 min CAP, black circles), expressed as percentages and depicted without CHX **(C)** and with CHX **(D)** as CAP-normalized regrowth curves. All results were depicted as medians with quartiles of six independent experiments in duplicate.

**FIGURE 4:**
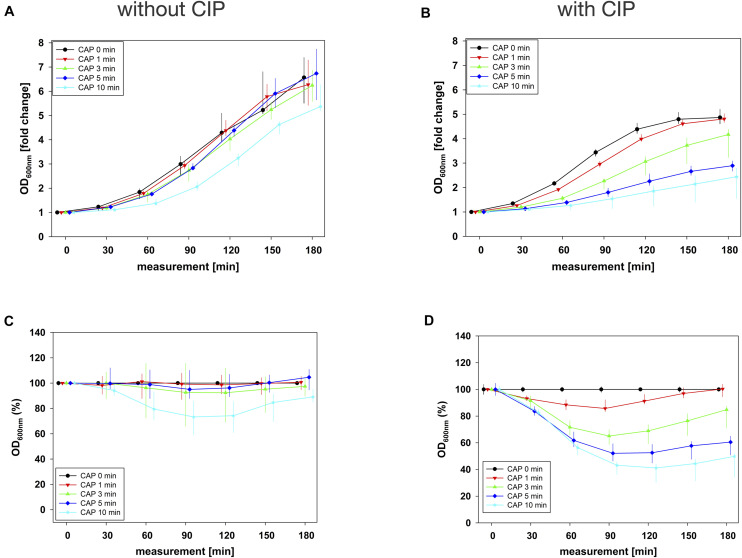
Antibacterial assay against *E. faecalis* biofilms with CAP alone and in binary combination with CIP first (sequence 1). Several CAP treatment periods were applied to *E. faecalis* biofilms and OD was measured at 600 nm every 30 min from 0 to 180 min after treatment. For each CAP treatment period, the values of measurement time = 0 were set to 1, corresponding following values were related to this and depicted as fold change. The results of the OD as a function of the measurement time were displayed as normalized regrowth curves **(A,B)**, those depicted in A were treated with CAP only, those in B were additionally treated with CIP (1 mg/mL, 10 min) first (sequence 1). These OD values were related to matching CAP-untreated samples for each measurement time (0 min CAP, black circles), expressed as percentages and depicted without CIP **(C)** and with CIP **(D)** as CAP-normalized regrowth curves. All results were depicted as medians with quartiles of six independent experiments in duplicate.

The original OD values of measurement time 0 min ranged between 0.1 and 0.2 in all combination experiments. These initial values were used for normalization and graphical depiction. In general, for all agents in all situations, the greatest effects were achieved by combining them with 10 min CAP (Figures 2–4 and Supplementary Figures S2–S4).

In combination experiments, sequence 1 was examined in which the biofilms were treated with CAP alone (0 mg/mL BAC, CHX or CIP) and in binary combination with BAC, CHX or CIP first. For BAC with 10 min CAP, no bacterial regrowth could be detected within the measurement period of 180 min, which was observed both in the normalized regrowth curve (Figure 2B) and in the CAP-normalized regrowth curve (Figure 2D). For CHX (Figure 3), the OD values of the combination at low CAP treatment periods were similar to those of the CAP treatment alone. For CIP (Figure 4), the greatest effects were already achieved with 5 min CAP.

**FIGURE 5:**
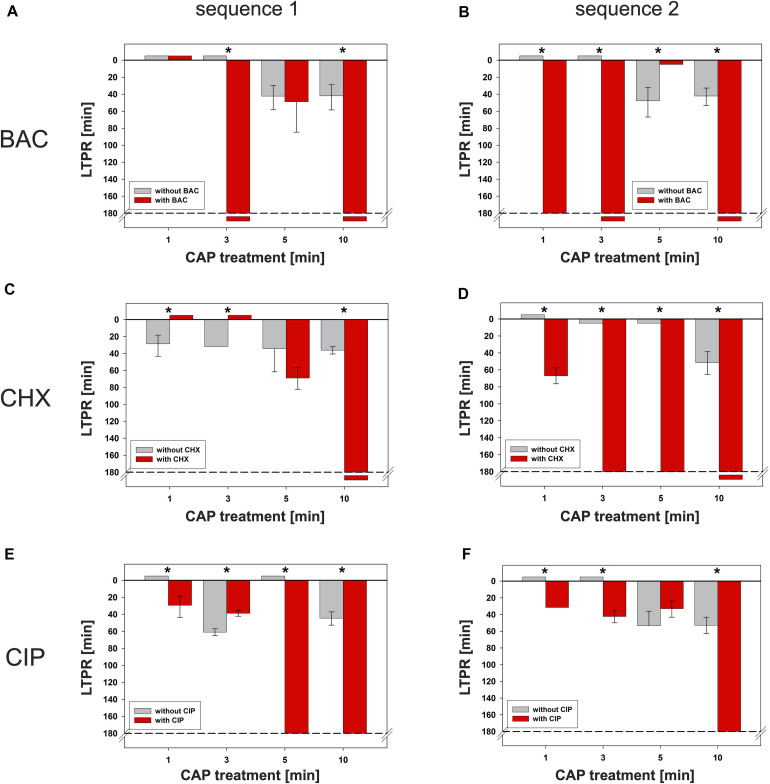
Latest time point of retreatment (LTPR) derived from CAP-normalized regrowth curves. Values of the CAP-normalized regrowth curves were fitted (resulting typically in dose response curves, Supplementary Figure S5), the first inflection points including 95% confidence intervals were derived and defined as LTPRs. The gray bars show the LTPR values of the CAP treatments alone, the red bars the binary combination of CAP with the corresponding agent. The LTPRs from the experiments with BAC (Figure 2 and Supplementary Figures S2C,D each) were shown in **(A)** (sequence 1) and **(B)** (sequence 2), those of CHX (Figure 3 and Supplementary Figures S3C,D each) and CIP (Figure 4 and Supplementary Figures S4C,D each) in **C–F**, respectively. Negative values mean that the graph runs like the reference line (0 min CAP), small positive values (5 min) mean that the corresponding fit was waveform shaped and therefore, in both cases no LTPRs were determinable. The dashed lines represent the end of the measurement period. The LTPR at 180 min means that the graph remains at its minimum level. If the LTPR is depicted later than 180 min, the graph decreases even further and no regrowth is detectable. The bars have been depicted downward to illustrate the flattening of the curve. For each treatment time asterisks denote significant differences (Tukey’s intervals) among CAP alone versus the combination with the respective agent. Values of this figure were repeatedly depicted as Table 1.

After that, sequence 2 was examined where the biofilms were treated with CAP alone and in binary combinations with BAC, CHX or CIP afterward. For BAC (Supplementary Figure S2), there was no effect in the results of the several CAP treatment periods compared to no CAP treatment (0 min CAP). For CHX (Supplementary Figure S3), larger differences between the several CAP treatment periods occurred at this sequence. For CIP (Supplementary Figure S4), only 10 min CAP prevented regrowth within the measurement period.

### Fits of CAP-Normalized Regrowth Curves

The fits of the CAP-normalized regrowth curves were the base for the calculated inflection points. The typical curve for a graph with an existing inflection point is exemplarily shown in Supplementary Figure S5A, where regrowth was delayed in comparison to the CAP-untreated sample at the beginning, and after the inflection point it increased again. The values here ranged between 70 and 100%, related to the CAP-untreated control. If no inflection point was derivable within the measurement period (exemplarily shown in Supplementary Figure S5B), the graph was irregular and the values here ranged between 97 and 104%, related to the CAP-untreated control.

### Latest Time Point of Retreatment (LTPR)

The time components of the first inflection points including 95% confidence intervals were derived from the fits as described above (Supplementary Figure S5A). The calculated inflection points which were defined as *latest time point of retreatment* (LTPR) were shown in Table 1 and in Figure 5. In general, treatments with CAP alone resulted in LTPRs in the range of 29 to 61 min. If no inflection point was derivable, no LTPR could be determined, for example if the graph ran approximately linear [e.g., CAP 1 min, Figure 2D and Table 1 (cells marked with ^§^)] or no regrowth was detectable during the measurement period of 180 min [e.g., CAP 10 min, Figure 2D and Table 1 (cells marked with ^#^)].

#### BAC (Figures 5A,B)

If CAP treatment was combined with BAC, the LTPR was at 180 min or even later. This was found at CAP treatment periods of at least 3 min or 1 min in the first or second sequence, respectively. Only the combination of BAC and 5 min CAP showed a lower LTPR at 49 min (sequence 1) or no determinable LTPR (sequence 2).

#### CHX (Figures 5C,D)

Due to the treatment with CHX first (sequence 1), the LTPR occurred not until 69 min (5 min CAP) or later than 180 min (10 min CAP). A CHX treatment afterward (sequence 2) led to the LTPR at 67 min after only 1 min CAP, while LTPRs were at 180 min or later for CAP treatment periods of 3 min or longer.

#### CIP (Figures 5E,F)

In sequence 1, the additional treatment with CIP resulted in LTPRs at 180 min after CAP treatment for 5 min or longer, while in sequence 2 LTPRs at 180 min were only found at CAP treatment of 10 min. At shorter CAP treatment periods, LTPRs were in the range of those found for the CAP single treatment.

## Discussion

The increasing development of resistance to conventional antibiotics and antiseptics in nosocomial pathogens like *E. faecalis* is a long-standing problem for public healthcare (Murray, 1990). Conventional antibacterial agents often no longer achieve the expected clinical success (Rossolini and Mantengoli, 2008), since especially the application of a single substance may only exert non-lethal effects on bacteria, for example organized in a biofilm. However, a combined binary application of non-lethal concentrations of two antibacterial strategies may show bactericidal or even disinfectant efficacy. This is why novel antibacterial approaches like binary treatments may be an option to prevent further spread of resistance and to eliminate such resistant bacteria successfully. Therefore, the aim of this study was to investigate the effect on antibacterial efficacy when binary combinations of conventional antibacterial agents with CAP, each in non-lethal concentrations, are applied.

The reactive species resulting from the production of CAP offer the advantage that they do not have a specific but several targets on the bacteria, which makes the development of resistances very unlikely (Zimmermann et al., 2012; Lunov et al., 2017). In their 2014 review, O’Connor et al. (2014) presented the wide range of potential applications for CAP in clinical environments and predicted that CAP could effectively complement established antimicrobial approaches.

Although bacteria in biofilms can be 100 to 1000 times more tolerant toward antimicrobial treatment than their planktonic counterparts (Ceri et al., 1999; Ronqui et al., 2016), it has already been shown that CAP can be successfully used for the inactivation of *E. faecalis* in biofilms (Li et al., 2015; Theinkom et al., 2019). However, since the antimicrobial efficacy of one single approach will reach its limits at some point, it may be useful to combine CAP with other antibacterial approaches. Combination therapies have the advantage that the risk of bacteria becoming resistant to both approaches simultaneously can be considered as very unlikely (Mouton, 1999). Furthermore, combination therapies may offer the potential to reduce concentrations of both agents or shorten the treatment periods of both approaches or even gain reductions in both, concentrations and treatment periods (Taraszkiewicz et al., 2013; Wozniak and Grinholc, 2018; Nakonieczna et al., 2019). Suchlike synergistic effects have already been shown for combination of CAP with CHX or CIP (Brun et al., 2018).

However, the focus of the present study was not on testing for synergy, but for evaluating the effect of a specific binary combination of CAP with an antimicrobial agent during the first 3 h period after application. Therefore, the regrowth of the treated and untreated cultures was measured every 30 min during a 3 h period starting immediately after treatment (0 min). Effective treatment results in no growth during this period, whereas weakened cultures first show a delayed growth, followed by an increase of growth, typically reaching growth like untreated controls. This idea was formally realized by deriving the inflection point of the CAP normalized regrowth curves. At the time component of this inflection point the growth of the culture turns from delayed to increasing, that means that at this point the growth potential of the culture is at its maximum. This time point was considered as the latest reasonable time point where a retreatment appears to be appropriate for preventing regrowth of the bacteria in case the first treatment was not lethal. Therefore, this time point was named *latest time point of retreatment* (LTPR).

First of all, range finding experiments were performed. They showed that the regrowth of *E. faecalis* treated with several CAP treatment periods was delayed as compared to the untreated control (Supplementary Figure S1A), but regrowth of the bacteria was still possible during the measurement period of 180 min upon treatment. In the experiments with BAC, CHX, and CIP (Supplementary Figures S1B–D) different concentrations were tested to determine non-lethal concentrations for the following combination experiments. Care was taken to ensure that the regrowth of the bacteria was delayed after treatment, while still sufficient regrowth was detectable.

In order to evaluate the effect on the antibacterial efficacy of the binary combination of CAP with BAC, CHX or CIP, the first inflection points of the CAP-normalized regrowth curves were determined and defined as LTPRs.

Within the given measurement period, these LTPRs provide the following possibilities for detecting antibacterial effects: (i) Single treatment vs. single treatment, (ii) single treatment vs. binary combination treatments and (iii) binary combination treatments vs. binary combination treatments. In each case, if no regrowth is detectable (antibacterial effect) or if the regrowth of the culture is similar to the untreated control (no effect), the LTPR does not exist. If LTPR exists, a higher value of treatment option A compared to treatment option B means that option A was more effective than option B. In this case, if the effect of the treatment was not lethal, the LTPR is a decisive characteristic time point. After the treatment, the surviving bacteria start to replicate again. Up to the LTPR, the rate of regrowth is increasing to its maximum, while thereafter it decreases. In order to prevent the bacteria from complete regrowth, a retreatment appears to be appropriate at the latest at the LTPR. Therefore, a higher LTPR value compared to another is more effective because the treatment has to be started again at a later time point.

Here in this study, an antibacterial effect was determined for example in sequence 1, BAC with CAP 10 min (Table 1 and Figure 5A). In sequence 2 for example, CHX with CAP 1 min (Table 1 and Figure 5D) showed a higher LTPR compared to the single treatment with CAP 1 min. This means that the combination of CAP with CHX was more effective compared to CAP without CHX. No effect was determined for example in sequence 1, BAC with CAP 1 min (Table 1 and Figure 5A).

In many studies, the results of binary treatments are presented by measuring colony forming units (CFU) after incubation for at least 24 h (Ronqui et al., 2016; Iluz et al., 2018). In contrast, the LTPR as outlined in the present study provides information about regrowth in the first 3 h upon treatment. By consecutively measuring OD of the resuspended bacteria in the period after the treatment, it can be determined if and when a retreatment may be necessary and appropriate to prevent regrowth of the bacteria. In addition, determining CFU-reduction rates at one single time point may be still worthwhile for fulfilling the requirements outlined by the American Society of Microbiology (ASM) for using the term “antimicrobial” (defined as reduction by 3 log_10_ steps of CFU as compared to untreated control) (Pearson et al., 1980; Taylor et al., 1983).

In this study, the results from the combination experiments using CAP with BAC, CHX or CIP, respectively, showed that the binary combinations can lead to significantly higher LTPRs than single treatments. In this case, the binary treatments are significantly more effective or even lead to antibacterial effects, if the regrowth within the measurement period is missing. In this study, we even found differences in the LTPR results of the two sequences and the respective antibacterial agents.

Since both BAC and CHX have a similar mode of action (Gilbert and Moore, 2005), similar results were to be expected. However, here the results vary depending on the agents used. Moreover, the sequences of the binary combinations showed an influence. Although the mode of action of BAC and CHX is a bit different in detail, the common target of the two agents is the cytoplasmic membrane (McDonnell and Russell, 1999; Gilbert and Moore, 2005). Cations such as Ca^2+^ normally stabilize the lipid bilayer of bacterial cytoplasmic membranes. When quaternary ammonium compounds like BAC or biguanides like CHX are applied, the cations are replaced by their positively charged head groups, which can bind to negatively charged phospholipids of the lipid bilayer (Gilbert and Moore, 2005; Cieplik et al., 2019). CHX carries two positive charges, which leads to a stronger binding than with BAC, which is single positively charged (Gilbert and Moore, 2005). With both agents, this mechanism disturbs the fluidity and creates hydrophilic gaps in the membrane. As a result, the membrane integrity is destroyed and there is leakage of cytoplasmic components. In contrast to BAC, which interacts with the entire membrane structure, CHX only acts superficially on the lipid bilayer (Gilbert and Moore, 2005).

These slight differences in the mechanism of action and structure of BAC or CHX may be reasons why the results found in this study are not exactly identical for both agents. There may also be differences in the ability of BAC or CHX to penetrate the biofilm matrix (extracellular polymeric substances, EPS). For instance, there may be differences in electrostatic interactions with negatively charged EPS-residues depending on the number of positive charges (Stewart, 2015). Furthermore, a preceding treatment with CAP could damage or modify the EPS to such an extent that CHX is able to penetrate more efficiently and thus shows a higher antimicrobial efficacy, while its double-positive charge might otherwise impede penetration.

In contrast to CHX and BAC, CIP as an antibiotic has a different mode of action by inhibiting the enzyme DNA gyrase. Therefore, it was expected that pre-damage by CAP (sequence 2) would lead to later LTPRs due to better accessibility of CIP to its target structure. However, the binary combination of CAP 5 min with CIP first (Table 1, sequence 1, CAP 5 min with CIP) results in a later LTPR than applying CIP afterward (Table 1, sequence 2, CAP 5 min with CIP). Because CIP inhibits the DNA synthesis of the bacteria and thus also cell division, it is particularly effective when the bacteria replicate rapidly. A previous CAP treatment might have already slowed bacterial replication to such an extent that CIP might be less effective.

Based on the method used in this study, it may be interesting for the future to investigate bacteria that exhibit resistance to one of the tested agents in order to get an idea which structures and mechanisms could be attacked by the binary treatment.

## Conclusion

The *latest time point of retreatment* (LTPR) is a novel method to evaluate the antibacterial efficacy of single or binary treatments with different antibacterial approaches, based on the bacterial regrowth curves upon treatment. The LTPR is defined as the time component of the inflection point of a normalized regrowth curve and allows the detection of antibacterial effects. If no regrowth is detectable (antibacterial effect), or if the regrowth of the culture is similar to the untreated control (no effect), the LTPR does not exist. Generally, if LTPR exists, a lower LTPR value of a given treatment option A compared to a given treatment option B means that option A was less effective than option B. In addition, LTPR designates the latest time point where a retreatment appears to be appropriate for preventing regrowth of the bacteria in case the first treatment was not lethal. Therefore, a lower LTPR value compared to another is less effective because the treatment has to be started again at an earlier time point.

The results of the combination experiments of non-lethal doses of BAC, CHX or CIP with CAP demonstrated that the binary combination treatments have significantly higher LTPRs than single treatments. In this case, the binary treatments are significantly more effective or even can lead to antibacterial effects, whereby the sequence of application is likely to have an influence. Thus, LTPR provides a new promising option to distinguish effects for single or binary use of antimicrobial approaches.

## Data Availability Statement

The authors confirm that the data supporting the findings of this study are available within the article and its Supplementary Material. Further inquiries and raw data that support the findings of this study are available from the corresponding author (TM), upon reasonable request.

## Author Contributions

SS: data collection-lead, data analyzing-lead, writing original draft, discussion-lead, and editing. K-AH: conceptualization-equal, formal analysis-lead, software-supporting, methodology-lead, and data validation-lead. SC and HW: data collection-supporting, investigation-supporting, technical support, discussion, and editing-supporting. MC: investigation-supporting, technical support, discussion, and editing-supporting. JZ: project administration, investigation-supporting, technical support, writing, and editing-supporting. FC: conceptualization, data validation, writing, discussion, and editing-supporting. TM: conceptualization-lead, supervision-lead, writing, and editing-lead. All authors contributed to the article and approved the submitted version.

## Conflict of Interest

The authors declare that the research was conducted in the absence of any commercial or financial relationships that could be construed as a potential conflict of interest.
