# Supplementary material for: The Latest Time Point of Retreatment (LTPR) as a Novel Method to Determine Antibacterial Effects for Binary Use of Cold Atmospheric Plasma and Conventional Agents
Source: Front Microbiol. 2020 Oct 29;11:576500. doi: 10.3389/fmicb.2020.576500 (PMC7658100; doi:10.3389/fmicb.2020.576500)
Supplement: Supplementary file 1 [file Data_Sheet_1.docx]

|  | **A**, CAP |  | **B**, BAC | |
| --- | --- | --- | --- | --- |
|  | 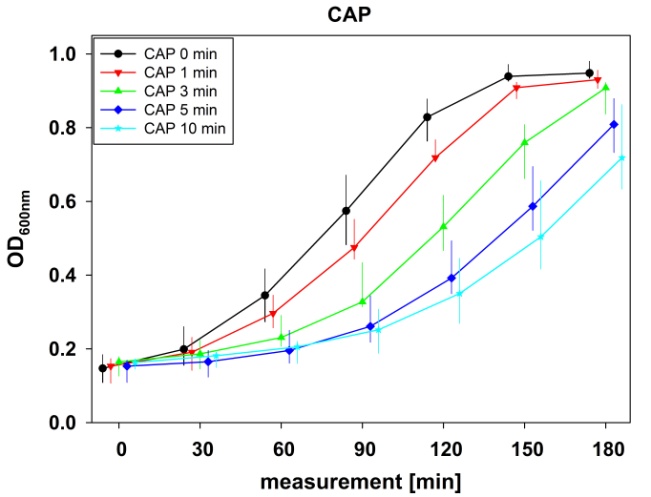 |  | 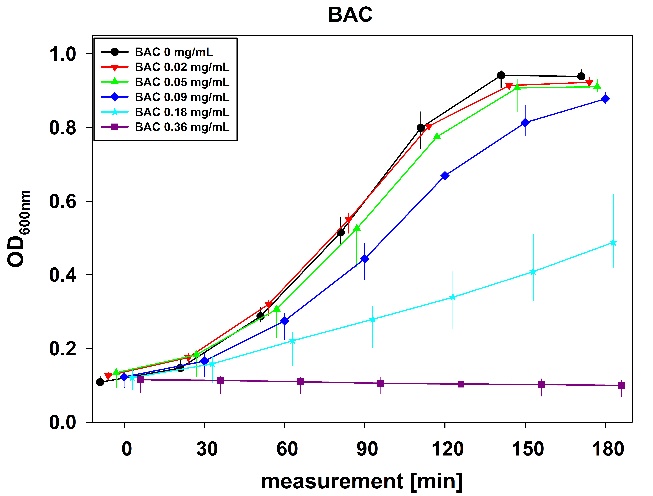 | |
|  |  |  |  | |
|  | **C**, CHX |  | **D**, CIP | |
|  | 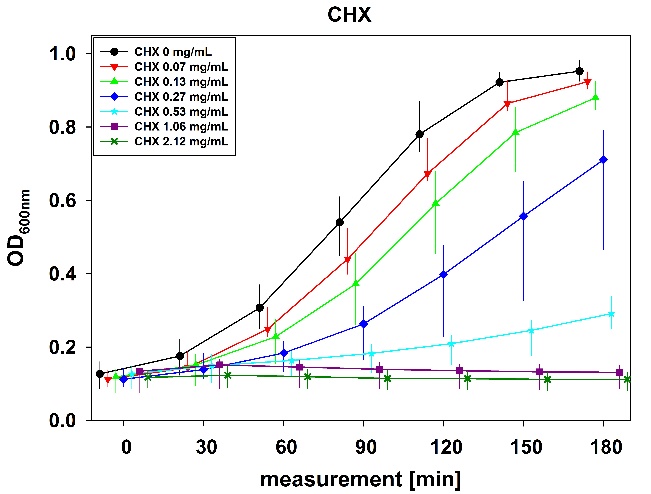 |  | 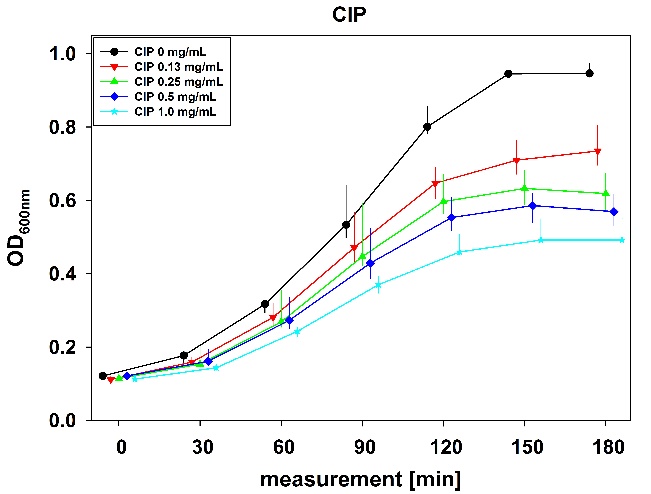 | |
| **Figure S1. Regrowth curves of the range finding experiments.** The OD of *E. faecalis* biofilms treated with several treatment periods of CAP (A) and different concentrations of BAC (B), CHX (C) and CIP (D) was measured at 600 nm every 30 min from 0 to 180 min after treatment. The selected sublethal concentrations for BAC, CHX and CIP in the combination experiments were 0.18 mg/mL, 0.27 mg/mL and 1 mg/mL, respectively. All results were depicted as medians with quartiles of six independent experiments in duplicate. | | | |  |

|  | | without BAC |  | with BAC | |
| --- | --- | --- | --- | --- | --- |
|  | **A** | |  | **B** | |
|  | 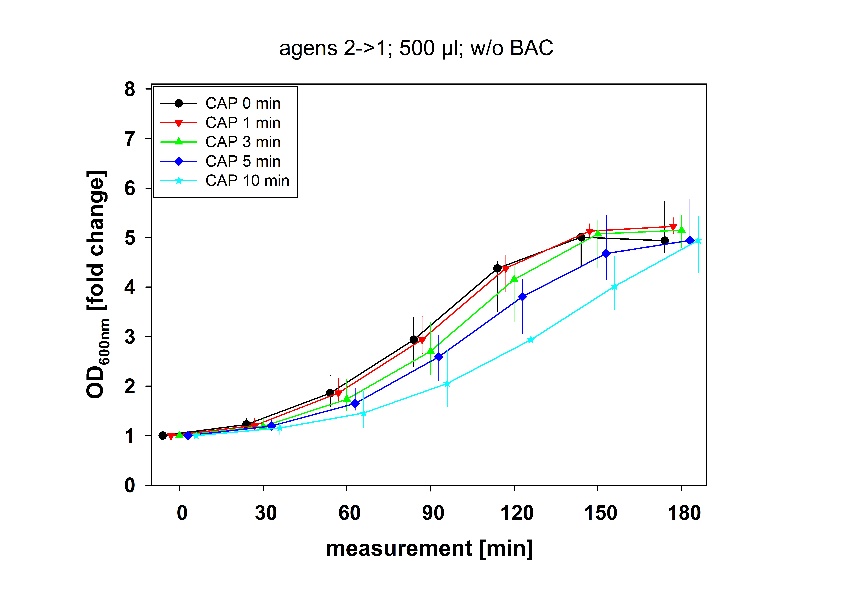 | |  | 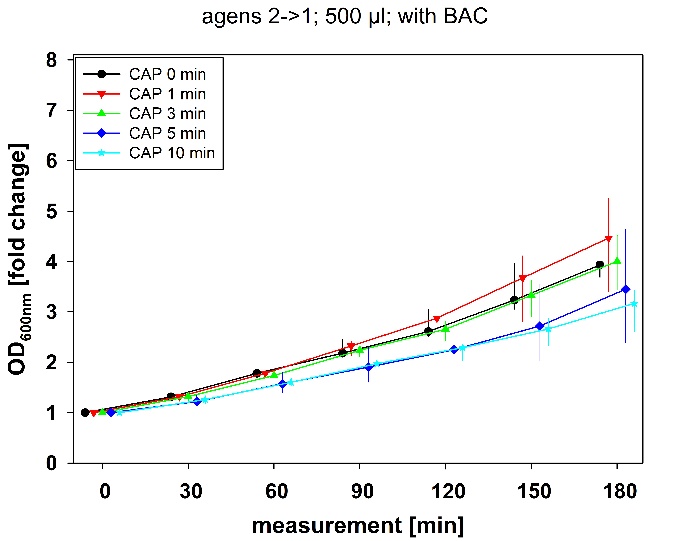 | |
|  | **C** | |  | **D** | |
|  | 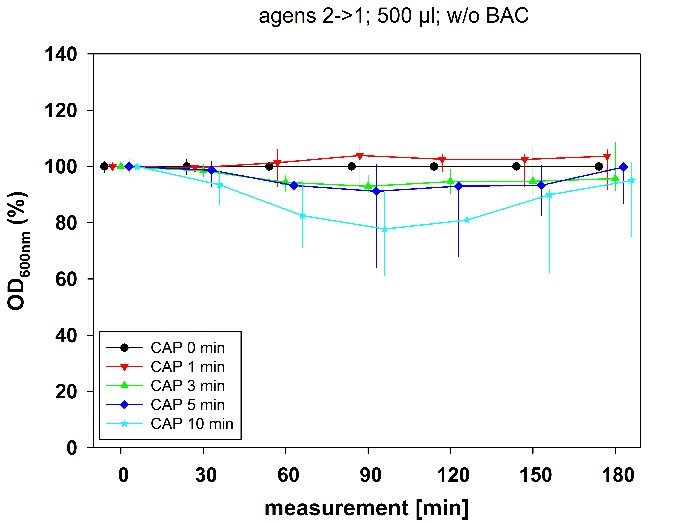 | |  | 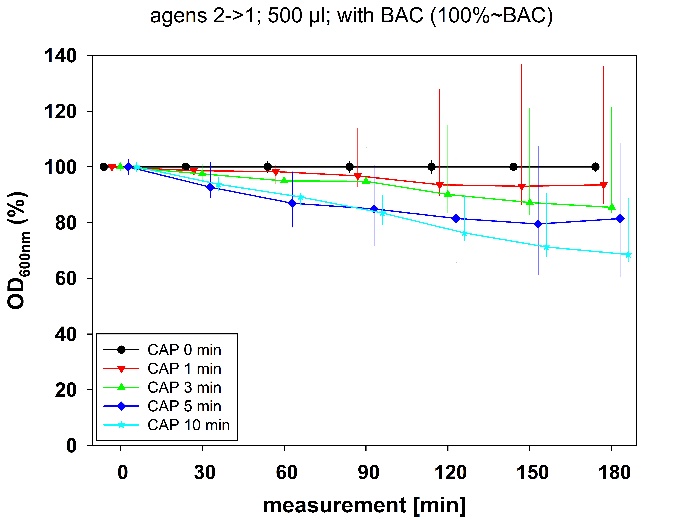 | |
| **Figure S2. Antibacterial assay against *E. faecalis* biofilms with CAP alone and in binary combination with BAC afterwards (sequence 2).** Several CAP treatment periods were applied to *E. faecalis* biofilms and OD was measured at 600 nm every 30 min from 0 to 180 min after treatment. For each CAP treatment period, the values of measurement time = 0 were set to 1, corresponding following values were related to this and depicted as fold change. The results of the OD as a function of the measurement time were displayed as normalized regrowth curves (A, B), those depicted in A were treated with CAP only, those in B were additionally treated with BAC (0.18 mg/mL, 5 min) afterwards (sequence 2). These OD values were related to matching CAP-untreated samples for each measurement time (0 min CAP, black circles), expressed as percentages and depicted without BAC (C) and with BAC (D) as CAP-normalized regrowth curves. All results were depicted as medians with quartiles of six independent experiments in duplicate. | | | | |  |

|  | | without CHX |  | with CHX | |
| --- | --- | --- | --- | --- | --- |
|  | **A** | |  | **B** | |
|  | 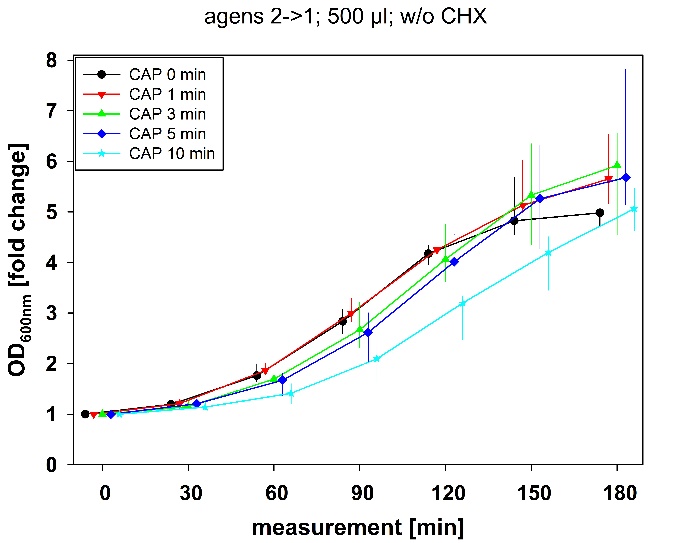 | |  | 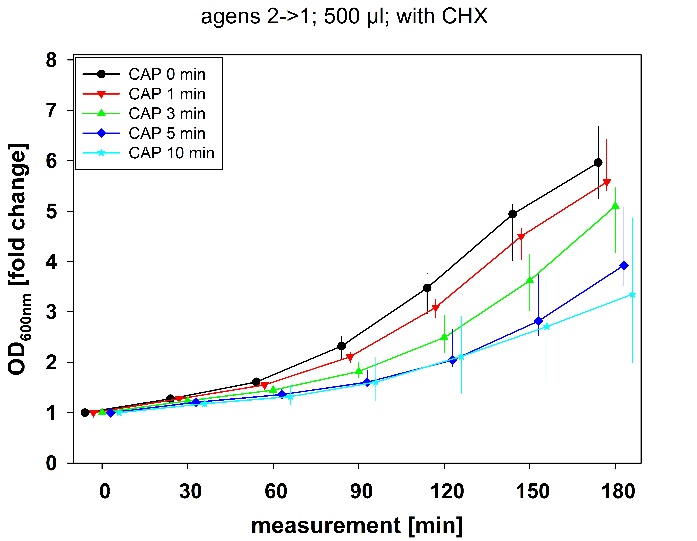 | |
|  | **C** | |  | **D** | |
|  | 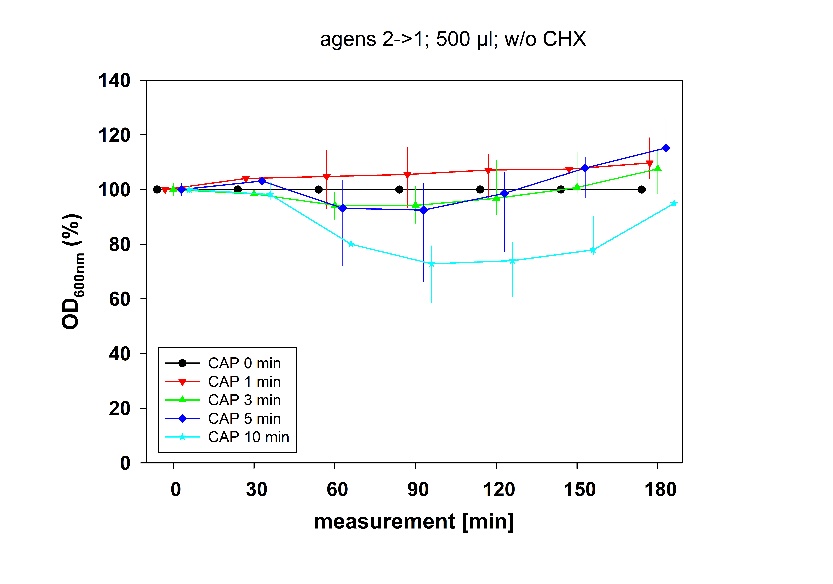 | |  | 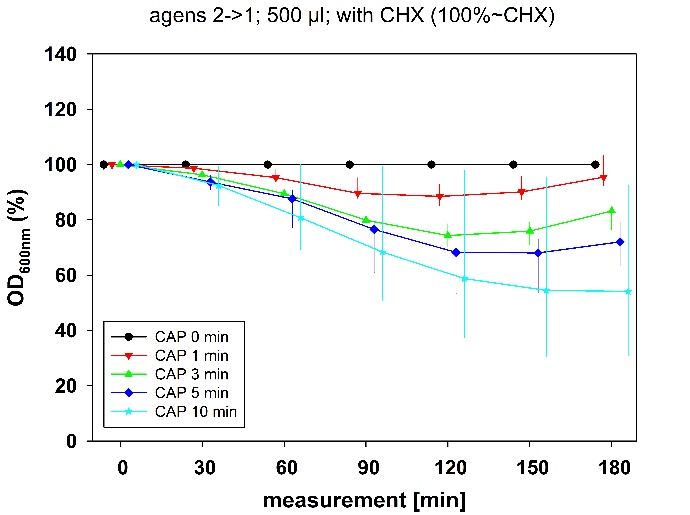 | |
| **Figure S3. Antibacterial assay against *E. faecalis* biofilms with CAP alone and in binary combination with CHX afterwards (sequence 2).** Several CAP treatment periods were applied to *E. faecalis* biofilms and OD was measured at 600 nm every 30 min from 0 to 180 min after treatment. For each CAP treatment period, the values of measurement time = 0 were set to 1, corresponding following values were related to this and depicted as fold change. The results of the OD as a function of the measurement time were displayed as normalized regrowth curves (A, B), those depicted in A were treated with CAP only, those in B were additionally treated with CHX (0.27 mg/mL, 1 min) afterwards (sequence 2). These OD values were related to matching CAP-untreated samples for each measurement time (0 min CAP, black circles), expressed as percentages and depicted without CHX (C) and with CHX (D) as CAP-normalized regrowth curves. All results were depicted as medians with quartiles of six independent experiments in duplicate. | | | | |  |

|  | | without CIP |  | with CIP | |
| --- | --- | --- | --- | --- | --- |
|  | **A** | |  | **B** | |
|  | 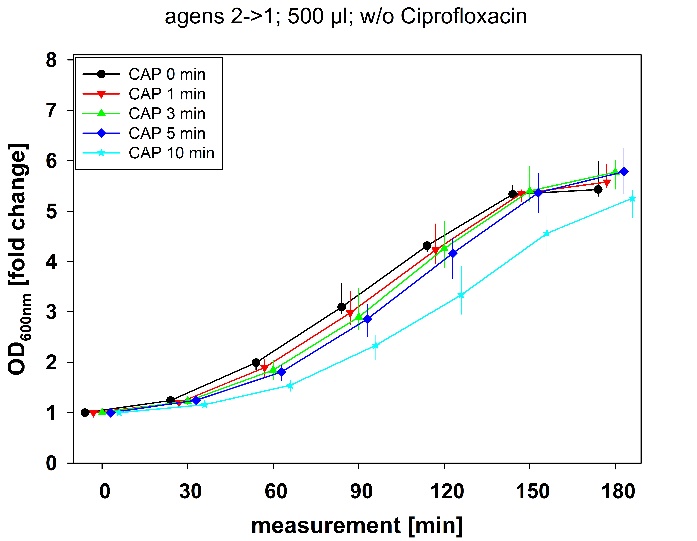 | |  | 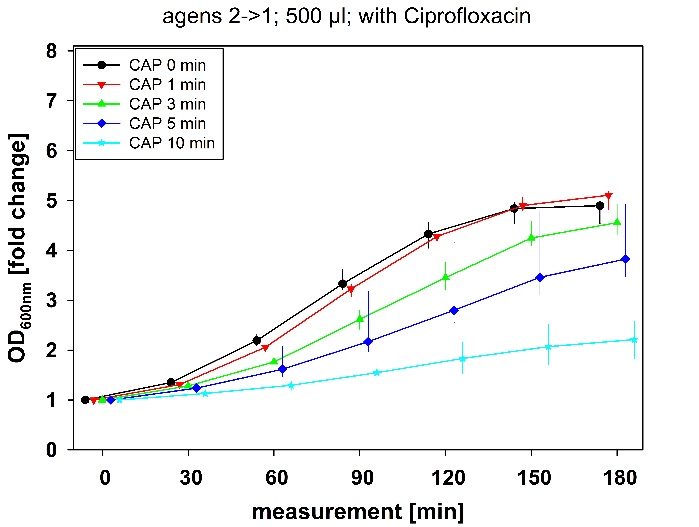 | |
|  | **C** | |  | **D** | |
|  | 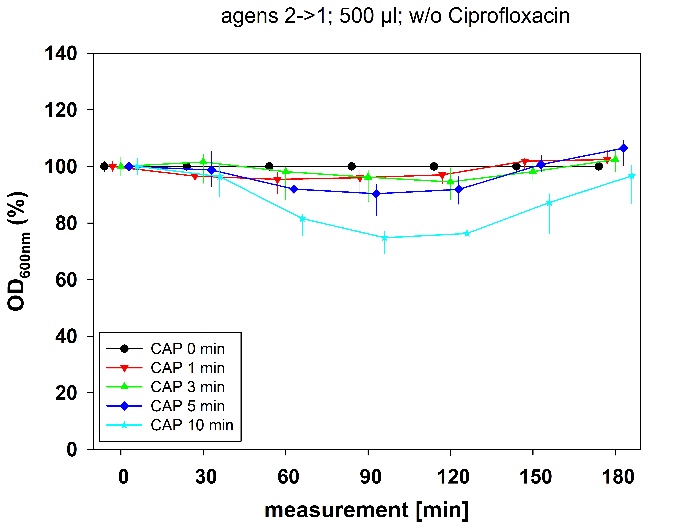 | |  | 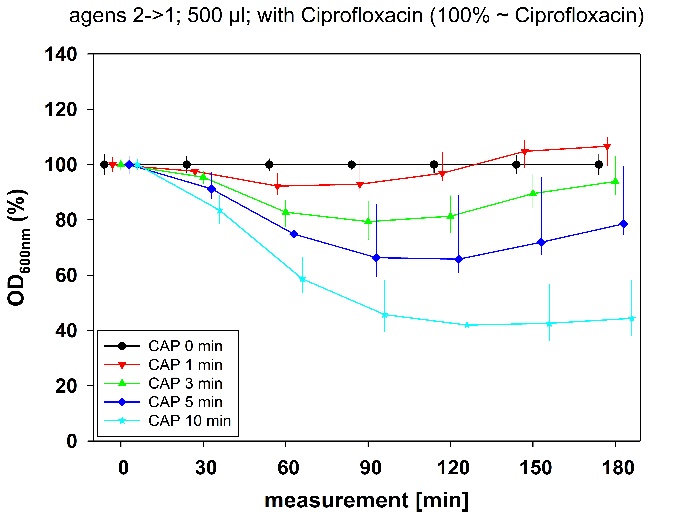 | |
| **Figure S4. Antibacterial assay against *E. faecalis* biofilms with CAP alone and in binary combination with CIP afterwards (sequence 2).** Several CAP treatment periods were applied to *E. faecalis* biofilms and OD was measured at 600 nm every 30 min from 0 to 180 min after treatment. For each CAP treatment period, the values of measurement time = 0 were set to 1, corresponding following values were related to this and depicted as fold change. The results of the OD as a function of the measurement time were displayed as normalized regrowth curves (A, B), those depicted in A were treated with CAP only, those in B were additionally treated with CIP (1 mg/mL, 10 min) afterwards (sequence 2). These OD values were related to matching CAP-untreated samples for each measurement time (0 min CAP, black circles), expressed as percentages and depicted without CIP (C) and with CIP (D) as CAP-normalized regrowth curves. All results were depicted as medians with quartiles of six independent experiments in duplicate. | | | | |  |

| **A** |  |
| --- | --- |
| 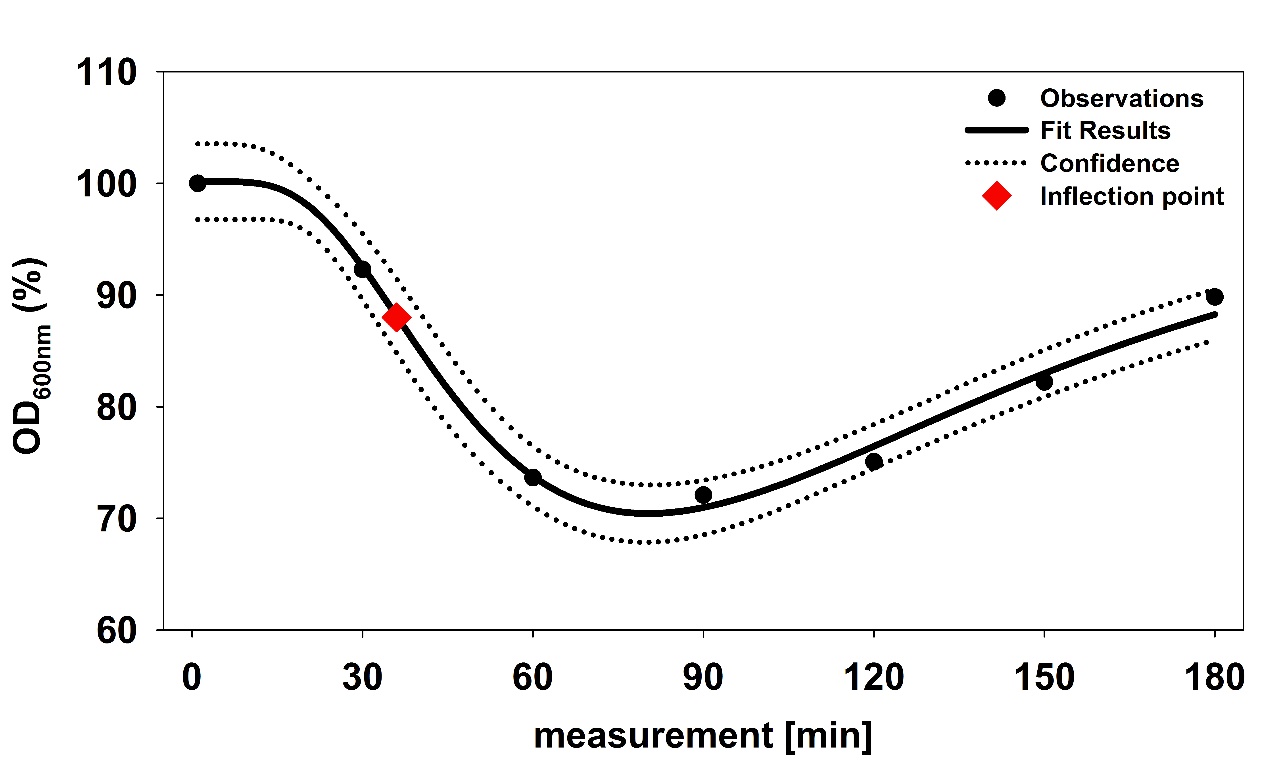 |  |

| **B** |
| --- |
| 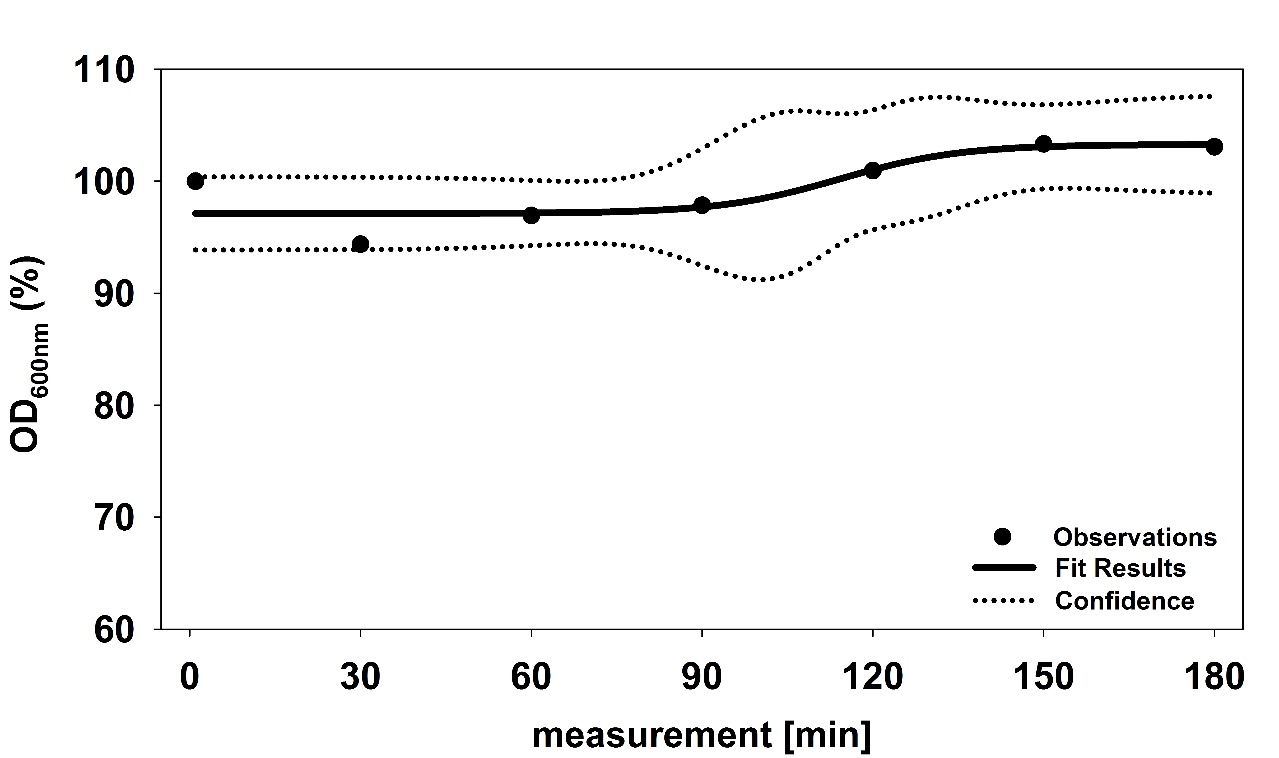 |
| **Figure S5. Examples for the fitted graphs.** Values of the CAP-normalized regrowth curves were fitted and depicted as dose response curves. **A** shows the typical shape when an inflection point was derivable. In this condition (sequence 1, CAP 10 min, without CHX) the inflection point (red diamond) was at 36 (32-41) min. **B** shows a characteristic graph of a condition (sequence 1, CAP 1 min, with BAC), where no inflection point was derivable. |
